# *BMC Ecology *embraces biodiversity research

**DOI:** 10.1186/1472-6785-10-16

**Published:** 2010-06-04

**Authors:** Hans Zauner

**Affiliations:** 1 BioMed Central, 236 Gray's Inn Road, London WC1X 8HL, UK

## Editorial

*BMC Ecology *has always been open to a wide range of topics from biodiversity research, and the International Year of Biodiversity [1] presents an opportunity to clarify that the journal includes subject matter that extends beyond the borders of ecology *sensu stricto*. We continue to consider all articles related to the ecology of animals, plants and microorganisms and wish to emphasise 'biodiversity' within the scope of the journal. We are confident that ecologists, evolutionary biologists and all scientists interested in biodiversity research will welcome our extended invitation to share this niche in an open access community journal.

It is also an appropriate time to acknowledge the synthesis of research traditions that is continuing under the umbrella of 'biodiversity research'. Biodiversity researchers come from all walks of academic life. They include systematic biologists, working with museum or herbarium specimens, who collect and describe new species and delineate others. Some academics may be branded as biodiversity researchers for their interest in evolutionary biology and speciation. Ecologists and conservation biologists, studying how species interact with each other and their environment, share a mutual goal in discovering how to protect threatened ecosystems. While interrelated, these different traditions have developed their own methods and to a certain extent their own language. 'Biodiversity research' unites these different research traditions. While some taxonomists would protest at being referred to as an ecologist (and vice versa), they can hardly object to being called biodiversity scientists. The input and collaboration of different research traditions is key, if progress is to be made in understanding life on earth in all its variety - and progress has to be made fast, with ecosystems under threat from causes too many and all too familiar.

What can a journal such as *BMC Ecology *do to promote collaboration of biodiversity researchers? And what sets *BMC Ecology *apart from other journals?

## All *BMC Ecology *content is open access

While most scientists and funding bodies now appreciate the value of open access publishing, the options for ecologists and biodiversity researchers wishing to publish open access in a trusted, highly visible, community journal are somewhat limited. This is surprising, and many argue that open access should be a more prominent issue in biodiversity and ecology [2]. We should consider the benefits of open access to Ecology and Biodiversity researchers:

### Open access comes with open data

Biodiversity researchers have to get to grips with enormous datasets, starting with the historic record of species descriptions accumulated since the time of Carl Linnaeus, to genomic information, barcoding initiatives, ecological surveys, satellite surveillance data, GPS tracking records, and more. A new discipline, biodiversity informatics, has a goal to develop tools and pipelines that let the scientist see the bigger picture emerging from these various types of data [3]. For this approach to be successful, all data has to be accessible to the research community without restrictions. The Biodiversity Heritage Library [4] is an initiative of libraries and research institutions which has started to digitise historic biodiversity literature. Scientists can enjoy easy access to the often groundbreaking work of their predecessors via an online repository and are fortunate that they can share their data much more easily with the global community than previous generations. *BMC Ecology *authors are encouraged to make raw data freely accessible and we don't have restrictions on the amount of additional material that can be deposited with our articles, which are all freely available from our homepage [5]. We also welcome initiatives that encourage standardized reporting and public deposition of datasets, such as the Global Biodiversity Information Facility [6], Encyclopedia of Life [7], and ZooBank [8]. Open access publishing is the best way that authors can ensure that their data will contribute to the bigger picture and have a lasting impact.

### Open access and society

To obtain continuing support for biodiversity research, and the conservation policies that emerge from it, scientists need to convince the public that it is a worthy endeavour and that they can be entrusted to make sensible use of taxpayers' money in times of decreasing financial resources. Biodiversity researchers need to be transparent about the process that leads to novel findings, especially if they have an impact on political decisions that affect all of us. Everyone should be able to scrutinize results of primary research on topics such as climate change and man-made biodiversity loss. Sure, many scientific papers are rather technical and not most people's favourite breakfast time reading. But unlike other disciplines of modern biology, biodiversity research is quite accessible to a wider audience. Examples of *BMC Ecology *articles of interest beyond scientific circles include a study by Lora Ghobrial and colleagues [9], who used genetic tools to track evidence for hunting and smuggling of Chimpanzees in Cameroon and, on a lighter note, an article by Nicolas Mathevon and colleagues [10], who deciphered the messages hyenas transmit in their laughing calls. Even more, the well-informed amateur always played an important role in biodiversity research, and continues to do so today. Ornithology, say, would be much worse off without the detailed records of enthusiastic and experienced amateur bird watchers. On a recent visit to a bird hide in the splendid London Wetlands Centre the most endemic of British species, the common bird watcher, was plainly visible. Listen carefully and you could overhear them discussing the genetics of bird migration, while sitting in front of their spotting scopes watching redshank and little-ringed plover wading through the mud. Research scientists should make better use of open access to reach out to this army of biodiversity enthusiasts supporting their cause.

### Open access and global cooperation

On May 15, the snake collection at the Butantan institute in Sao Paolo, Brazil, caught fire, destroying one of the world's largest collections of venomous animals [11]. The institute housed around 85.000 specimen of snakes, together with more than 450 000 spiders and scorpions. 90 years of research had been lost in one day. For the Brazilian scientists, and for researchers all over the world, this was a devastating blow, but this sad event reminds us of another distinguishing feature of biodiversity research: Biodiversity research is a global enterprise, and centres of excellence are not confined to the nations with traditionally large budgets for science. Researchers in developing countries profit from the spirit of open access data sharing in two ways. Wherever they are, they have unrestricted and immediate access to biodiversity data gathered in other corners of the world, and they can share their data with the international community by publishing in highly visible open access journals. BioMed Central, like other open access publishers, supports research in the developing world [12], where biodiversity resources are at high risk and cost much to conserve.

In summary, biodiversity research has all the ingredients to have a big future: a real urgency (that's the gloomy bit), but also the ability to engage people and unite them; new tools to connect and visualize millions of individual data points, and the disappearance of boundaries between previously more isolated disciplines. Publishing in open access journals can accelerate this potential, and with our new emphasis on biodiversity, *BMC Ecology *is ready to play its part in the exciting times ahead.
